# Association of treatment-emergent symptoms identified by patient-reported outcomes with adjuvant endocrine therapy discontinuation

**DOI:** 10.1038/s41523-022-00414-0

**Published:** 2022-04-21

**Authors:** Karen Lisa Smith, Neha Verma, Amanda L. Blackford, Jennifer Lehman, Kelly Westbrook, David Lim, John Fetting, Antonio C. Wolff, Daniela Jelovac, Robert S. Miller, Roisin Connolly, Deborah K. Armstrong, Raquel Nunes, Kala Visvanathan, Carol Riley, Katie Papathakis, Nelli Zafman, Jennifer Y. Sheng, Claire Snyder, Vered Stearns

**Affiliations:** 1 Johns Hopkins Women’s Malignancies Program, Johns Hopkins University School of Medicine, Baltimore, MD USA; 2 Johns Hopkins Department of Medicine, Johns Hopkins University School of Medicine, Baltimore, MD USA; 3 Division of Biostatistics and Bioinformatics, Johns Hopkins Sidney Kimmel Comprehensive Cancer Center, Baltimore, MD USA; 4 Division of Cancer Epidemiology, Johns Hopkins Bloomberg School of Public Health, Baltimore, MD USA; 5 Department of Health Policy and Management, Johns Hopkins Bloomberg School of Public Health, Baltimore, MD USA; 6 Department of Oncology, Johns Hopkins University School of Medicine, Baltimore, MD USA; 7 Present Address: Duke Cancer Institute, Duke University Medical Center, Durham, NC USA; 8 Present Address: CancerLinQ, American Society of Clinical Oncology, Alexandria, VA USA; 9 Present Address: Cancer Research @UCC, College of Medicine and Health, University College Cork, Cork, Ireland

**Keywords:** Breast cancer, Targeted therapies, Breast cancer

## Abstract

Many patients discontinue endocrine therapy for breast cancer due to intolerance. Identification of patients at risk for discontinuation is challenging. The minimal important difference (MID) is the smallest change in a score on a patient-reported outcome (PRO) that is clinically significant. We evaluated the association between treatment-emergent symptoms detected by worsening PRO scores in units equal to the MID with discontinuation. We enrolled females with stage 0-III breast cancer initiating endocrine therapy in a prospective cohort. Participants completed PROs at baseline, 3, 6, 12, 24, 36, 48, and 60 months. Measures included PROMIS pain interference, fatigue, depression, anxiety, physical function, and sleep disturbance; Endocrine Subscale of the FACT-ES; and MOS-Sexual Problems (MOS-SP). We evaluated associations between continuous PRO scores in units corresponding to MIDs (PROMIS: 4-points; FACT-ES: 5-points; MOS-SP: 8-points) with time to endocrine therapy discontinuation using Cox proportional hazards models. Among 321 participants, 140 (43.6%) initiated tamoxifen and 181 (56.4%) initiated aromatase inhibitor (AI). The cumulative probability of discontinuation was 23% (95% CI 18–27%) at 48 months. For every 5- and 4-point worsening in endocrine symptoms and sleep disturbance respectively, participants were 13 and 14% more likely to discontinue endocrine therapy respectively (endocrine symptoms HR 1.13, 95% CI 1.02–1.25, p = 0.02; sleep disturbance HR 1.14, 95% CI 1.01–1.29, *p* = 0.03). AI treatment was associated with greater likelihood of discontinuation than tamoxifen. Treatment-emergent endocrine symptoms and sleep disturbance are associated with endocrine therapy discontinuation. Monitoring for worsening scores meeting or exceeding the MID on PROs may identify patients at risk for discontinuation.

## Introduction

Although 5–10 years of adjuvant endocrine therapy reduces recurrence and death after early hormone receptor-positive (HR + ) breast cancer, approximately 50% of patients are non-adherent (do not take endocrine therapy as prescribed) or non-persistent (discontinue endocrine therapy early)^1–14^. While some patients discontinue shortly after initiation, others do so later, with persistence overall declining with time^15,16^. Risks of recurrence and death are higher among those who are non-adherent or who discontinue endocrine therapy early^8,17–19^.

Common symptoms during endocrine therapy include musculoskeletal discomfort, sleep disturbance, fatigue, anxiety, depression, and endocrine symptoms, such as vaginal dryness and hot flashes^10,12,20–30^. Side effects are frequently cited as a reason for early endocrine therapy discontinuation and multiple studies have demonstrated associations between symptoms and non-adherence or discontinuation^9,10,12,16,23,24,26,27,29,31–45^. However, prospective identification of patients at risk for early discontinuation due to intolerance remains a clinical challenge.

Symptoms experienced by patients during endocrine therapy are often under-appreciated by clinicians^46^. Patient-reported outcomes (PRO) are assessments from patients about their health status without interpretation by a clinician^47^. The minimal important difference (MID) is the smallest change in a score on a PRO measure that patients perceive as beneficial or harmful and that would affect management^48^. Prior studies evaluating the association of symptoms with endocrine therapy discontinuation have not used changes in PRO scores meeting or exceeding the MID to identify clinically important symptoms.

We report findings from a clinic-based cohort of women receiving endocrine therapy for early HR + breast cancer who completed PRO measures over 5 years. We aimed to evaluate the association of treatment-emergent symptoms, defined as worsening scores compared to baseline in increments equal to the MID for each PRO measure, with discontinuation of endocrine therapy prior to completing 5 years of treatment. We hypothesized that patient-reported new or worsening symptoms could identify individuals at risk for early discontinuation.

## Results

### Participant characteristics

Of 321 participants, 140 (43.6%) initiated tamoxifen and 181 (56.4%) an aromatase inhibitor (AI). Seventeen (5.3%) participants received ovarian function suppression (OFS) and 6 (1.9%) enrolled upon switching endocrine therapy agents. The median age at enrollment was 63 years and 65.4% were post-menopausal. The majority of participants were White (83.5%) and lived in zip codes with low neighborhood poverty (85.9%). Participants who initiated tamoxifen were younger than those who initiated an AI (Table 1).

**Table 1:** Characteristics of study population according to type of endocrine therapy initiated at study enrollment.

Characteristic	Overall Study Population *N* = 321	Tamoxifen *N* = 140	Aromatase Inhibitor *N* = 181
Median (Q1-Q3) age in years	63 (56–71)	54 (49–59)	69 (64–74)
Post-menopausal – N (%)	210 (65.4)	34 (24.3)	176 (97.2)
Ovarian Function Suppression – N (%)	17 (5.3)	16 (11.4)	1 (0.6)
Switching Endocrine Therapy^a^ – N (%)	6 (1.9)	0 (0)	6 (3.3)
Race – N (%)			
White	268 (83.5)	112 (80)	156 (86.2)
Black	33 (10.3)	16 (11.4)	17 (9.4)
Other	20 (6.2)	12 (8.6)	8 (4.4)
Neighborhood Poverty Rate^b^ – N (%)			
0–15%	274 (85.9)	116 (82.9)	158 (88.3)
>15%	45 (14.1)	24 (17.1)	21 (11.7)
Median number of concomitant medications at enrollment (range)	4 (0–29)	3 (0–29)	5 (0–22)
Stage – N (%)			
0	28 (8.7)	18 (12.9)	10 (5.5)
I	191 (59.5)	80 (57.1)	111 (61.3)
II	79 (24.6)	39 (27.9)	40 (22.1)
III	23 (7.2)	3 (2.1)	20 (11)
ER-positive – N (%)^c^	320 (100)	139 (100)	181 (100)
PR-positive – N (%)^c^	282 (88.7)	128 (93.4)	154 (85.1)
HER2-positive – N (%)	26 (8.9)	10 (8.2)	16 (9.4)
Mastectomy – N (%)	143 (44.5)	76 (54.3)	67 (37)
Radiation – N (%)	215 (67)	85 (60.7)	130 (71.8)
Chemotherapy – N (%)	90 (28.2)	43 (30.9)	47 (26.1)
Median duration of follow-up in months (range)^d^	56.1 (6.9–87.7)	57.9 (9.1–87.7)	54.4 (6.9–87.3)

*Q1-Q3* interquartile range, *ER* estrogen receptor, *PR* progesterone receptor, *HER2* human epidermal growth factor receptor-2, *SD* standard deviation.

^a^ Participants were eligible to enroll at the time they first initiated adjuvant endocrine therapy or at the time of switching from one type of adjuvant endocrine therapy to another.

^b^ Neighborhood poverty rate is the percentage of persons living in a zip code with a family income below the federal poverty line based on United States census data. Neighborhood poverty rate was missing for two participants.

^c^ ER status was missing for one participant. PR status was missing for 3 participants.

^d^ Follow-up was calculated as the time from study entry to last clinic visit or last PRO survey completion, whichever came last.

### Scores on PRO measures

**Fig. 1:**
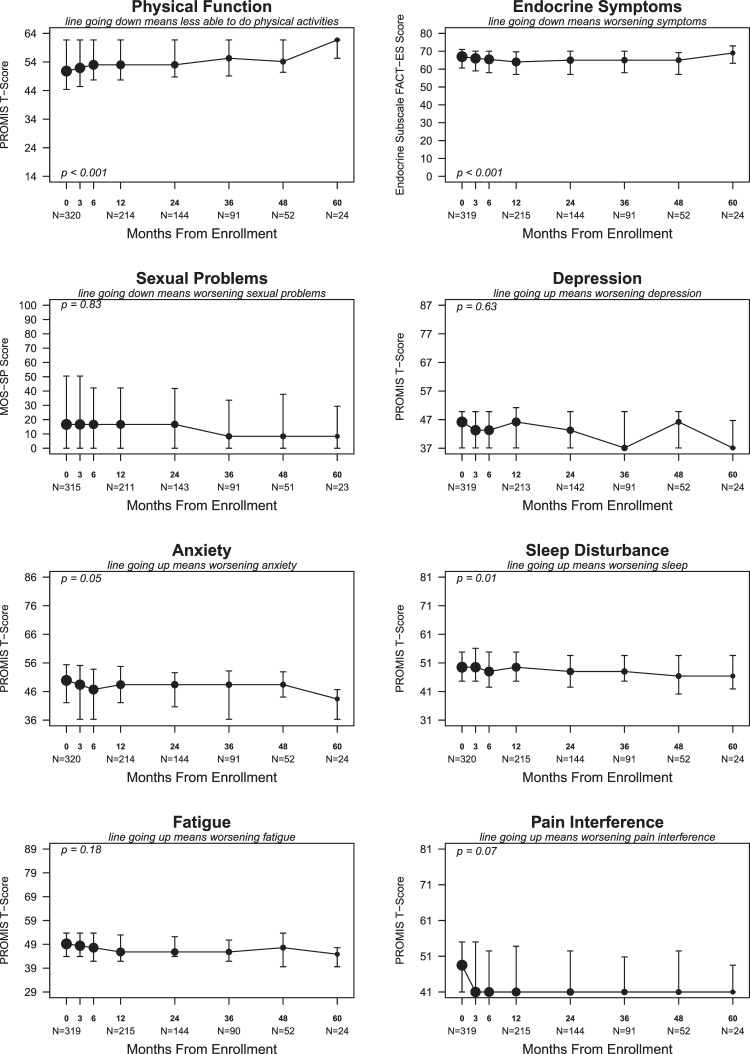
Change in patient-reported outcome scores over time. Line graphs display median PRO scores at each time point. The size of each dot is proportional to the number of participants who completed the PRO measure at that time point. The numbers of participants who completed the PRO measure at baseline and at 12, 24, 36, 48, and 60 months point are noted under the X-axis at the corresponding time points. The Y-axis denotes the score range for each PRO measure. Bars represent interquartile ranges. *P*-values summarize overall mean change in PRO scores during the first 24 months compared to baseline with a four-degree-of-freedom test. PRO patient-reported outcomes.

**Fig. 2:**
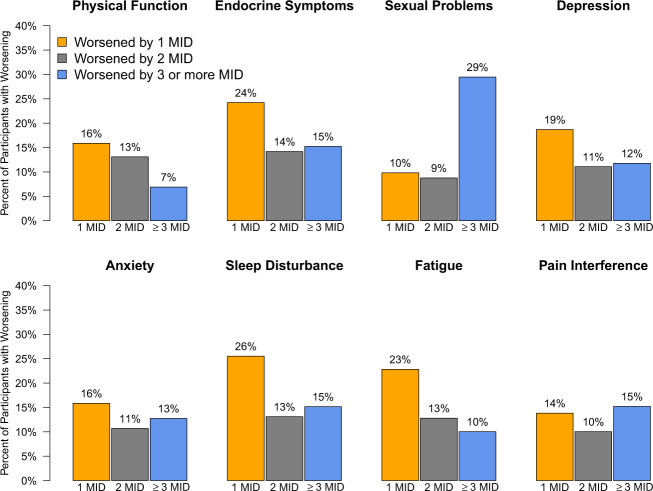
Proportion of participants who experienced worsening of patient-reported outcome scores exceeding the minimal important difference at any time point through 60 months. Bar plots display worst change in PRO scores at any time point in follow-up through 60 months. Worst changes are categorized as at least the MID but less than twice the MID, at least twice the MID but less than three times the MID and at least three times the MID for each measure. Only participants with baseline values and at least one follow-up measure are included. The proportions of participants whose worst changes in each PRO scores were less than the MID (i.e. who experienced improvement, no change or worsening less than the MID) are not displayed. The MID was considered to be 4 points for the physical function, depression, anxiety, sleep disturbance, fatigue, and pain interference measures; 8 points for the sexual problems measure; and 5 points for the endocrine symptoms measure. MID minimal important difference, 1 MID at least the MID but less than twice the MID, 2 MID twice the MID but less than three times the MID, 3 MID at least three times the MID, PRO patient-reported outcomes.

**Fig. 3:**
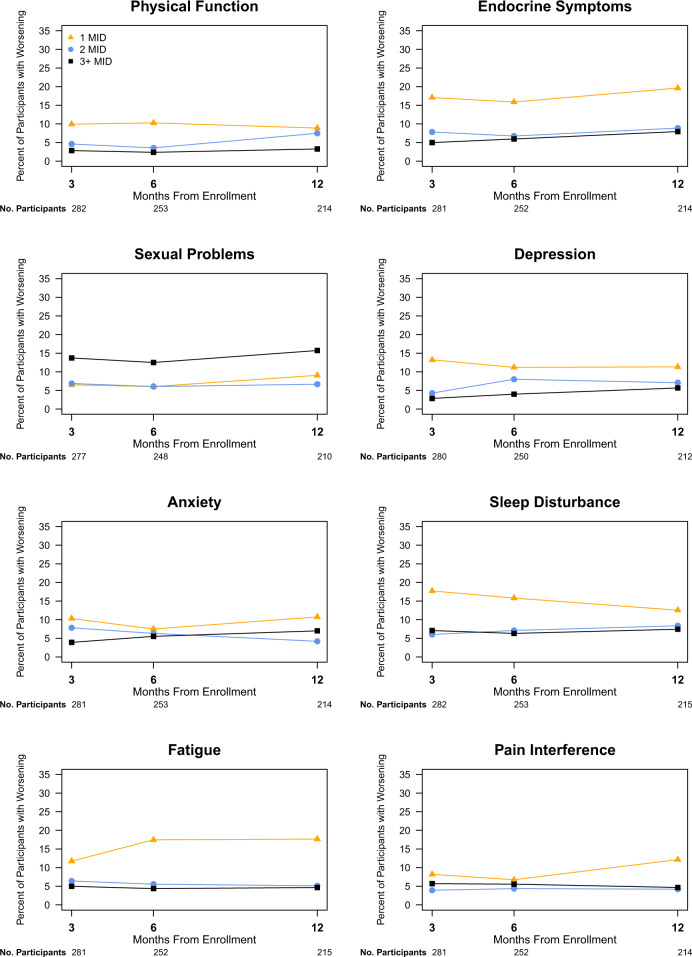
Proportion of participants who experienced worsening of patient-reported outcome scores meeting or exceeding the minimal important difference at 3, 6, and 12 months. Line graphs display percentage of participants with worsening of PRO scores compared to baseline at 3, 6, and 12 months after enrollment. Worsening of PRO scores is categorized as at least the MID but less than twice the MID, at least twice the MID but less than three times the MID and at least three times the MID for each measure. Only participants with baseline values and at least one follow-up measure are included. The percentage of participants whose PRO scores worsened by less than the MID (i.e. who experienced improvement, no change or worsening less than the MID) are not displayed. The MID was considered to be 4 points for the physical function, depression, anxiety, sleep disturbance, fatigue, and pain interference measures; 8 points for the sexual problems measure; and 5 points for the endocrine symptoms measure. MID minimal important difference, 1 MID at least the MID but less than twice the MID, 2 MID twice the MID but less than three times the MID, 3+ MID at least three times the MID, PRO patient-reported outcomes.

Mean scores for all PRO measures were within one SD of published population means at all time points. The proportion of participants who completed the PROs declined over time (Table 2). Mean changes in PRO scores during the first 24 months were small, and, while statistically significant for worsening endocrine symptoms (*p* < 0.001), the mean (SD) change in the FACT-ES score from baseline to 24 months was −2.9 (8.3), which is less than 1 MID (Fig. 1). Despite small mean changes, the worst change in the score at any time up to 60 months met or exceeded the MID for each measure in over one-third of participants. Symptom domains with the greatest proportions of participants with score worsening meeting or exceeding the MID at any time up to 60 months were sleep disturbance (54%), endocrine symptoms (53%), sexual problems (48%), and fatigue (46%) (Fig. 2). Treatment-emergent symptoms often developed soon after endocrine therapy initiation (Fig. 3). For example, worsening compared to baseline by at least the MID for sleep disturbance, endocrine symptoms, sexual problems, and fatigue was already observed at 3 months in 27.1%, 26.3%, 23.3%, and 20.3% of participants respectively (Table 3).

**Table 2:** Mean scores on patient-reported outcome measures at each study time point.

Domain	Baseline N^a^ Mean (SD)	3 months N^a^ Mean (SD)	6 months N^a^ Mean (SD)	12 months N^a^ Mean (SD)	24 months N^a^ Mean (SD)	36 months N^a^ Mean (SD)	48 months N^a^ Mean (SD)	60 months N^a^ Mean (SD)
Physical Function	320 51.5 (8.3)	282 52 (8.1)	253 53.3 (7.9)	214 53.2 (8.1)	144 53.9 (8.1)	91 54.5 (7.6)	52 54.3 (7.3)	24 56.8 (8.1)
Endocrine Symptoms	319 65.1 (8.6)	281 63.7 (8.8)	252 63.7 (8.4)	214 62.5 (9.4)	143 62.9 (8.9)	90 63.4 (8.1)	52 63.4 (8.2)	24 67.2 (7.5)
Sexual Problems	315 27.4 (32.0)	277 27.9 (33.7)	248 25.8 (31.4)	210 27.1 (31.1)	141 25.2 (30.2)	90 21.6 (28)	51 25.3 (30.1)	23 20.4 (29.8)
Depression	319 45.4 (7.8)	280 44.9 (8.1)	250 44.6 (8)	212 45.2 (8.1)	142 44.2 (8.2)	91 43.5 (7.8)	52 44.9 (6.3)	24 43.0 (7.6)
Anxiety	320 49.0 (9.4)	281 47.9 (9.1)	253 47.5 (8.7)	214 48.1 (8.6)	144 47.4 (8.4)	91 47.3 (8.5)	52 47.6 (7.5)	24 43.8 (7.5)
Sleep Disturbance	320 49.0 (8.1)	282 49.8 (8.7)	253 48.8 (8.3)	215 49.1 (8.65)	144 47.6 (8.3)	91 49 (7.8)	52 46.8 (9)	24 43.8 (7.5)
Fatigue	319 48.7 (7.7)	281 48.3 (7.9)	252 48 (8.5)	215 47.3 (8.3)	146 46.4 (8.9)	90 46.6 (7.5)	52 46.1 (7.9)	24 43.3 (5.9)
Pain Interference	319 48.4 (8.2)	281 47.7 (8.2)	252 46.8 (7.6)	214 47.4 (7.5)	143 47.3 (7.7)	90 46 (6.7)	52 45.8 (7.4)	24 44.4 (5.6)

*SD* standard deviation.

^a^ N at each time point for each domain represents the number of participants who completed the patient-reported outcome measure.

**Table 3:** Change in symptoms compared to baseline at each time point through 12 months.

Symptom Domain	3 months *N* (%)	6 months *N* (%)	12 months *N* (%)
Physical Function^a^			
No change or improvement	189 (58.9)	184 (57.3)	150 (46.7)
Worsening by less than the MID	44 (13.7)	28 (8.7)	22 (6.9)
Worsening by at least the MID but less than twice the MID	28 (8.7)	26 (8.1)	19 (5.9)
Worsening by at least twice the MID but less than three times the MID	13 (4)	9 (2.8)	16 (5)
Worsening by at least three times the MID	8 (2.5)	6 (1.9)	7 (2.2)
PRO not completed	38 (11.8)	67 (20.9)	106 (33)
Endocrine Symptoms^b^			
No change or improvement	123 (38.3)	106 (33)	79 (24.6)
Worsening by less than the MID	74 (23.1)	74 (23.1)	57 (17.8)
Worsening by at least the MID but less than twice the MID	48 (15)	40 (12.5)	42 (13.1)
Worsening by at least twice the MID but less than three times the MID	22 (6.9)	17 (5.3)	19 (5.9)
Worsening by at least three times the MID	14 (4.4)	15 (4.7)	17 (5.3)
PRO not completed	39 (12.1)	68 (21.2)	106 (33)
Sexual Problems^c^			
No change or improvement	201 (62.6)	187 (58.3)	114 (44.9)
Worsening by less than the MID	1 (0.3)	0 (0)	0 (0)
Worsening by at least the MID but less than twice the MID	18 (5.6)	15 (4.7)	19 (5.9)
Worsening by at least twice the MID but less than three times the MID	19 (5.9)	15 (4.7)	14 (4.4)
Worsening by at least three times the MID	38 (11.8)	31 (9.7)	33 (10.3)
PRO not completed	43 (13.4)	72 (22.4)	110 (34.3)
Depression^a^			
No change or improvement	192 (59.8)	175 (54.5)	139 (43.3)
Worsening by less than the MID	31 (9.7)	17 (5.3)	22 (6.9)
Worsening by at least the MID but less than twice the MID	37 (11.5)	28 (8.7)	24 (7.5)
Worsening by at least twice the MID but less than three times the MID	12 (3.7)	20 (6.2)	15 (4.7)
Worsening by at least three times the MID	8 (2.5)	10 (3.1)	12 (3.7)
PRO not completed	40 (12.5)	70 (21.8)	108 (33.6)
Anxiety^a^			
No change or improvement	180 (56.1)	170 (53)	136 (42.4)
Worsening by less than the MID	39 (12.1)	34 (10.6)	31 (9.7)
Worsening by at least the MID but less than twice the MID	29 (9)	19 (5.9)	23 (7.2)
Worsening by at least twice the MID but less than three times the MID	22 (6.9)	16 (5)	9 (2.8)
Worsening by at least three times the MID	11 (3.4)	14 (4.4)	15 (4.7)
PRO not completed	39 (12.1)	67 (20.9)	106 (33)
Sleep Disturbance^a^			
No change or improvement	146 (45.5)	139 (43.3)	120 (37.4)
Worsening by less than the MID	49 (15.3)	40 (12.5)	34 (10.6)
Worsening by at least the MID but less than twice the MID	50 (15.6)	40 (12.5)	27 (8.4)
Worsening by at least twice the MID but less than three times the MID	17 (5.3)	18 (5.6)	18 (5.6)
Worsening by at least three times the MID	20 (6.2)	16 (5)	16 (5)
PRO not completed	38 (11.8)	67 (20.9)	105 (32.7)
Fatigue^a^			
No change or improvement	158 (49.2)	130 (40.5)	116 (36.1)
Worsening by less than the MID	58 (18.1)	53 (16.5)	40 (12.5)
Worsening by at least the MID but less than twice the MID	33 (10.3)	44 (13.7)	38 (11.8)
Worsening by at least twice the MID but less than three times the MID	18 (5.6)	14 (4.4)	11 (3.4)
Worsening by at least three times the MID	14 (4.4)	11 (3.4)	10 (3.1)
PRO not completed	39 (12.1)	68 (21.2)	105 (32.7)
Pain Interference^a^			
No change or improvement	209 (65.1)	193 (60.1)	153 (47.7)
Worsening by less than the MID	22 (6.9)	17 (5.3)	16 (5)
Worsening by at least the MID but less than twice the MID	23 (7.2)	17 (5.3)	26 (8.1)
Worsening by at least twice the MID but less than three times the MID	11 (3.4)	11 (3.4)	9 (2.8)
Worsening by at least three times the MID	16 (5)	14 (4.4)	10 (3.1)
PRO not completed	39 (12.1)	68 (21.2)	106 (33)

*MID* minimal important difference, *PRO* patient-reported outcome.

^a^ MID = 4 points;

^b^ MID = 5 points;

^c^ MID = 8 points.

Due to the decline in PRO completion rates over time, we performed a sensitivity analysis comparing participants who completed all measures during the first 24 months to those with at least one missing measure during that timeframe. With the exception of differences in the number of concomitant medications and mean baseline fatigue and physical function scores, those with and without missing measures had similar key baseline characteristics. The differences in mean baseline fatigue and physical function between these groups were less than the MID (Table 4).

**Table 4:** Characteristics of participants with and without missing patient-reported outcome measures during the first 24 months of study participation.

Characteristic	No Missing PRO Measures During First 24 Months (*N* = 132)	≥1 Missing PRO Measure During First 24 Months (*N* = 189)	*p* value^f^
Mean age in years (SD)	63.0 (10.5)	62.2 (11.3)	0.51
Post-menopausal – N (%)	85 (64.4)	125 (66.1)	0.81
Ovarian Function Suppression – N (%)	6 (4.5)	11 (5.8)	0.80
Endocrine Therapy			
Tamoxifen	64 (48.5)	76 (40.2)	0.17
AI	68 (51.5)	113 (59.8)	
Switching Endocrine Therapy^a^ – N (%)	0 (0)	6 (3.2)	0.05
Race – N (%)			
White	117 (88.6)	151 (79.9)	0.12
Black	9 (6.8)	24 (12.7)	
Other	6 (4.5)	14 (7.4)	
Neighborhood Poverty Rate^b^ – N (%)			
0–15%	117 (89.3)	157 (83.5)	0.19
>15%	14 (10.7)	31 (16.5)	
Median number of concomitant medications at enrollment (range)	4 (0–13)	5 (0–29)	0.006
Stage – N (%)			
0	15 (11.4)	13 (6.9)	0.13
I	78 (59.1)	113 (59.8)	
II	34 (25.8)	45 (23.8)	
III	5 (3.8)	18 (9.5)	
ER-positive – N (%)	132 (100)	189 (100)	
PR-positive^c^ – N (%)	110 (83.8)	172 (92.5)	0.02
HER2-positive^d^ – N (%)	8 (6.8)	18 (10.2)	0.40
Mastectomy – N (%)	59 (44.7)	84 (44.4)	>0.99
Radiation – N (%)	85 (64.4)	130 (63.8)	0.47
Chemotherapy^e^ – N (%)	36 (27.5)	54 (28.7)	0.90
Mean Baseline Depression Score (SD)	44.6 (7.5)	46.0 (8.1)	0.12
Mean Baseline Physical Function Score (SD)	52.7 (8.3)	50.0 (8.1)	0.005
Mean Baseline Endocrine Symptoms Score (SD)	65.8 (7.7)	64.6 (9.2)	0.21
Mean Baseline Sexual Problems Score (SD)	25.3 (29.7)	29.0 (33.6)	0.30
Mean Baseline Depression Score (SD)	44.6 (7.5)	46.0 (8.1)	0.12
Mean Baseline Anxiety Score (SD)	48.5 (9.2)	49.4 (9.5)	0.41
Mean Baseline Sleep Disturbance Score (SD)	48.2 (7.7)	49.6 (8.3)	0.13
Mean Baseline Fatigue Score (SD)	47.3 (8.0)	49.6 (7.4)	0.01
Mean Baseline Pain Interference Score (SD)	47.5 (7.9)	49.0 (8.3)	0.09
Discontinuation Status			
Completed Treatment – N (%)	24 (18.2)	2 (1.1)	<0.001
Discontinued due to Distant Metastases – N (%)	3 (2.3)	8 (4.2)	
Discontinued due to Locoregional Recurrence– N (%)	2 (1.5)	0 (0)	
Discontinued due to Side effects/Intolerance – N (%)	15 (11.4)	48 (25.4)	
Discontinued Tamoxifen to Transition to AI – N (%)	2 (1.5)	3 (1.6)	
Discontinued due to Other reasons – N (%)	2 (1.5)	8 (4.2)	
Still on Endocrine Therapy – N (%)	84 (63.6)	120 (63.5)	

*ER* estrogen receptor, *PR* progesterone receptor, *HER2* human epidermal growth factor receptor-2, *SD* standard deviation, *AI* aromatase inhibitor.

^a^ Participants were eligible to enroll at the time they first initiated adjuvant endocrine therapy or at the time of switching from one type of adjuvant endocrine therapy to another.

^b^ Neighborhood poverty rate is the percentage of persons living in a zip code with a family income below the federal poverty line based on United States census data. Neighborhood poverty rate was unknown for one participant with no missing PRO measures during the first 24 months and for one participant with at least one missing PRO measure during the first 24 months.

^c^ PR status was unknown for 3 participants with missing PRO measures during the first 24 months.

^d^ HER2 status was unknown for 15 participants with no missing PRO measures during the first 24 months and for 13 participants with at least one missing PRO measure during the first 24 months.

^e^ Receipt of chemotherapy was unknown for 1 participant with no missing PRO measures during the first 24 months and for 1 participant with at least one missing PRO measure during the first 24 months.

^f^ *P*-values are for Fisher’s exact test for categorical measures, *t*-tests for comparison of means and Wilcoxon rank sum tests for comparison of medians

### Cumulative probability of discontinuation

**Fig. 4:**
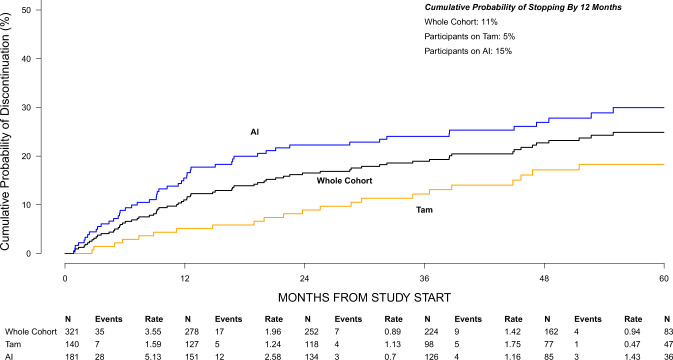
Cumulative probability of endocrine therapy discontinuation. Curves depict time to discontinuation of endocrine therapy for the entire cohort and according to type of endocrine therapy. AI aromatase inhibitor, Tam tamoxifen.

Median follow-up was 56.1 months and 204 (63.6%) participants remained on endocrine therapy at the time of data cut-off. Twenty-six (8.1%) participants had discontinued after completing the planned course and 13 (4.0%) due to recurrence. Five (1.6%) switched from the endocrine therapy initiated at enrollment to another agent with less than 6 weeks interruption. Sixty-three (19.6%) participants had stopped endocrine therapy due to side effects/intolerance and 10 (3.1%) due to other reasons besides recurrence, new primary breast cancer, completion of at least 5 years of endocrine therapy or switching agents. Among the 73 participants who discontinued due to side effects/intolerance or other reasons besides recurrence, new primary breast cancer, completion of at least 5 years of endocrine therapy or switching agents, the times at which they discontinued were distributed across the follow-up period. Ten discontinued in the first 3 months (13.7%), 10 in months 3–6 (13.7%), 6 in months 6–9 (8.2%), 9 in months 9–12 (12.3%), 9 in months 12–18 (12.3%), 8 in months 18–24 (11%), and 21 after 24 months (28.8%). Among participants with and without missing PRO measures in the first 24 months, 25.4% and 11.4% respectively discontinued due to side effects/intolerance (Table 4). The cumulative probabilities of discontinuation at 3, 6, 12, 24, 36, and 48 months were 3% [95% confidence interval (CI): 1–5%], 6% (95% CI: 4–9%), 11% (95% CI: 7–14%), 17% (95% CI: 12–21%), 19% (95% CI: 14–23%), and 23% (95% CI: 18–27%), respectively. Figure 4 depicts the time to discontinuation of endocrine therapy for the entire cohort and according to the type of endocrine therapy.

### Association of patient-reported outcomes and clinico-demographic factors with endocrine therapy discontinuation

Of the 73 study participants who discontinued endocrine therapy due to side effects/intolerance or due to other reasons besides recurrence, completion of at least 5 years of endocrine therapy or switching agents, 63 completed the PRO measures at the time point immediately before their discontinuation. And, among the 248 participants who did not discontinue endocrine therapy, 190 completed the PRO measures at the time point immediately before they completed follow-up. Therefore, we had complete data for evaluation of the association of PROs with time to discontinuation on 253 (79%) of patients.

In univariate analyses, worsening of multiple symptoms (endocrine symptoms, fatigue, sleep disturbance, and pain interference) plus receipt of AI were associated with endocrine therapy discontinuation. In the final multivariate model, treatment-emergent endocrine symptoms (adjusted HR 1.13, 95% CI 1.02 – 1.25, *p* = 0.02) and sleep disturbance (adjusted HR 1.14, 95% CI 1.01–1.29, *p* = 0.03) remained significantly associated with endocrine therapy discontinuation. Additionally, patients receiving AI were twice as likely to discontinue compared to those on tamoxifen (adjusted HR 1.98, 95% CI 1.17–3.33, *p* = 0.01). Higher stage was associated with lower likelihood of discontinuation (adjusted HR 0.61, 95% CI: 0.43 – 0.87, *p* = 0.006) (Table 5).

**Table 5:** Univariate and multivariate associations of new or worsening symptoms and clinico-demographic variables with time to discontinuation of adjuvant endocrine therapy.

Variable	Univariate Hazard Ratio (95% CI)	p-value	Multivariate Adjusted Hazard Ratio^a^ (95% CI)	p-value
Physical Function (4-point worsening)	1.06 (0.95–1.19)	0.30		
Endocrine Symptoms (5-point worsening)	1.15 (1.06–1.26)	0.001	1.13 (1.02–1.25)	0.02
Fatigue (4-point worsening)	1.15 (1.02–1.29)	0.02		
Depression (4-point worsening)	1.03 (0.93–1.15)	0.55		
Anxiety (4-point worsening)	1.05 (0.95–1.16)	0.33		
Sleep Disturbance (4-point worsening)	1.18 (1.06–1.32)	0.002	1.14 (1.01–1.29)	0.03
Sexual Problems (8-point worsening)	1.05 (1.00–1.11)	0.06		
Pain Interference (4-point worsening)	1.11 (1.00–1.24)	0.05		
Age in years	1.02 (1.00–1.04)	0.09		
Race (White vs. Other)	1.30 (0.67–2.53)	0.44		
High neighborhood poverty rate	1.10 (0.58–2.09)	0.78		
Adjuvant endocrine therapy (AI vs. Tamoxifen)	1.93 (1.17–3.19)	0.01	1.98 (1.17–3.33)	0.01
Number of baseline concomitant medications	1.05 (1.00–1.11)	0.07		
Higher Stage	0.71 (0.51–1.00)	0.05	0.61 (0.43–0.87)	0.006
HER2-positive	0.53 (0.17–1.70)	0.29		
Mastectomy	0.91 (0.57–1.45)	0.69		
Radiation	0.98 (0.60–1.59)	0.93		
Chemotherapy	0.47 (0.25–0.88)	0.02		

*AI* aromatase inhibitor, *CI* confidence interval, *HER2* human epidermal growth factor receptor-2.

^a^ Multivariable HR only shown for variables included in final model.

## Discussion

In this real-world prospective clinic-based cohort of women with early HR + breast cancer receiving endocrine therapy who completed PRO measures over 5 years, we demonstrated that treatment-emergent symptoms, defined as worsening scores at any time during follow-up compared to baseline, was associated with risk for discontinuing endocrine therapy prior to completing 5 years of treatment. Specifically, new or worsening endocrine symptoms and sleep disturbance were associated with endocrine therapy discontinuation. As the severity of the treatment-emergent symptoms worsened, the risk of discontinuation increased. For every 5-point worsening on the Endocrine Subscale of the FACT-ES measure, the risk of discontinuation was 13% higher, and for every 4-point worsening on the PROMIS sleep disturbance measure, the risk of discontinuation was 14% higher. Worsening of scores meeting or exceeding the MID of on these measures was common, each occurring in approximately half the study population, with approximately half of these participants experiencing worsening by more than twice the MID.

Although multiple factors may contribute to an individual patient’s decision to discontinue endocrine therapy, side effects are a key driver of this decision^49^. To date, interventions to support adherence and persistence have largely focused on educational or behavioral strategies and have had limited success^7,50^. We argue that early intervention to mitigate side effects has the potential to enhance endocrine therapy adherence and persistence. The cornerstone to this strategy, however, is comprehensive detection of treatment-emergent symptoms that may drive patients to discontinue therapy. Unfortunately, in routine clinical care, clinicians often underappreciate side effects patients experience during endocrine therapy, and the symptom burden reported by patients often exceeds that detected by clinicians^22,46^. Our data demonstrate that clinically relevant treatment-emergent symptoms are common during endocrine therapy and that use of PRO measures as an adjunct to routine clinical care can identify symptoms associated with early discontinuation.

When utilizing PRO measures in clinical care, the optimal score thresholds beyond which clinical action should be taken are uncertain and may vary depending on the clinical scenario, symptoms, specific measures, and clinical outcomes to which score thresholds are anchored^51,52^. It is possible that MIDs may vary by factors such as age, race, ethnicity, socioeconomic status, stage, treatment duration or intervention and, moreover, that MIDs may change over time. Although severity thresholds can identify particularly bothersome symptoms and absolute scores on PROs during the course of endocrine therapy have been associated with discontinuation, worsening of scores over time can indicate changes in supportive care needs and thus present a clinical opportunity to provide symptom management^9,53–56^. Our findings demonstrate that treatment-emergent symptoms, defined by score worsening compared to baseline meeting or exceeding the MID for the selected measure, can identify clinically significant symptoms associated with endocrine therapy discontinuation. Patients in whom these symptoms are identified should be targeted for enhanced symptom management with the dual goals of improving symptoms and supporting persistence.

Our findings build on previous work demonstrating that treatment-emergent symptoms often present soon after endocrine therapy initiation, indicating that symptom monitoring with PROs has the potential to identify patients at risk for discontinuation early during the course of therapy to whom interventions could be targeted^12,26,31^. However, as has also previously been reported, endocrine therapy discontinuation in our cohort increased over time and worsening of symptoms meeting or exceeding the MID at *any* time during 5 years of follow-up was associated with discontinuation^15,16,33,45^. Most prior studies have focused on associations between baseline symptoms or those that emerge early during endocrine therapy with discontinuation^9,10,12,24,26,27,31,32,37,42^. Our study is unique in that it demonstrates that treatment-emergent symptoms at any time over 5 years are associated with endocrine therapy discontinuation, supporting ongoing PRO monitoring throughout the course of therapy.

In our study, the cumulative probabilities of endocrine therapy discontinuation at 6, 12, 24, 36, and 48 months, respectively were 6, 11, 17, 19, and 23%. These observed discontinuation rates are lower than reported in many prior studies^8,11^. It is possible that completing PRO measures motivated patients to continue endocrine therapy. Alternatively, although we did not mandate that clinicians review scores nor implement symptom management interventions, it is possible that greater patient and clinician awareness of symptoms due to the PROs led to better symptom management and, in turn, to enhanced persistence.

Our findings are consistent with prior studies linking symptoms prior to or during receipt of tamoxifen or an AI such as endocrine symptoms and sleep disturbance with non-adherence and early treatment discontinuation^10,23,26,27,31,40,42,57^. Endocrine symptoms include vasomotor symptoms, weight changes, vaginal or sexual symptoms, mood changes and joint pain^30^. In patients with breast cancer, endocrine symptoms, such as hot flashes and night sweats, can disrupt sleep^58,59^. Evidence-based strategies can mitigate the treatment-emergent symptoms that were associated with early discontinuation of endocrine therapy in our cohort. For example, improved sleep hygiene, exercise, and cognitive behavioral therapy can support patients with sleep disturbance^60–67^. For patients with hot flashes or sweats, medications such as anti-depressants or gabapentin may improve both daytime symptoms and sleep^68,69^.

As has been previously reported, endocrine therapy discontinuation was more frequent among participants in our study taking an AI than tamoxifen, a finding potentially attributable to frequent musculoskeletal discomfort during AI therapy^32,70,71^. We also confirmed previous findings demonstrating that individuals with higher stage disease are less likely to discontinue endocrine therapy, a finding potentially explained by greater motivation to take therapy in light of higher recurrence risk^33,35,44^. In addition to PRO scores, these factors may guide identification of patients at risk for endocrine therapy discontinuation for interventions to support persistence.

Strengths of our study are that we comprehensively assessed common symptoms during endocrine therapy prospectively over 5 years with validated PRO measures in a real-world population. Many previous studies evaluating symptoms during endocrine therapy used cross-sectional or retrospective designs and, of those that were prospective, many did not use validated measures, assessed fewer symptoms or limited symptom assessment to early during the course of therapy^10,12,16,23,26,27,31–33,37,40–42,57,72^.

A key limitation of our study is that the proportion of participants who completed the PROs declined over time. In our sensitivity analysis, baseline characteristics of participants with and without missing measures were similar with the exception of the number of baseline concomitant medications. Some literature demonstrates an association between polypharmacy and endocrine therapy discontinuation, however, this association has not been consistently demonstrated and no prior literature indicates that a difference in one drug (the difference we observed in the median number of concomitant medications for those with and without missing PROs in the first 24 months) is meaningful^73–75^. Thus, we believe our sensitivity analysis indicates patients with and without missing PRO measures are similar and that our findings are robust despite missing data. Of note, however, the proportion of participants who discontinued endocrine therapy due to side effects/intolerance was higher among participants with missing measures suggesting that patients with greater symptom burden may have been less likely to complete the PROs. If participants with greater symptoms did not complete the PROs, it is possible that the association between treatment-emergent symptoms and endocrine therapy discontinuation is even stronger than we estimated. But, the fact that PRO data was complete for assessing the time to discontinuation for 79% of study participants supports our findings regarding the association between treatment-emergent symptoms and endocrine therapy discontinuation. The extent of missing PRO data in our study speaks to the need for strategies to further engage patients in incorporating PROs into their care. Considerations may include reducing the length or frequency of assessments to limit respondent burden and assessing PROs in conjunction with clinic visits.

Another limitation of our study is that few pre-menopausal participants received OFS and an AI, potentially limiting generalizability of our findings to the many young patients who may receive this therapy in light of recently published survival data. Additionally, few participants enrolled upon switching endocrine therapies. Although some patients who switch due to side effects tolerate the second agent, our findings cannot fully address tolerance after switching^10^. Additionally, we grouped patients taking tamoxifen and an AI, however, the treatment-emergent symptoms that identify those at risk for discontinuation may differ by drug. It is also possible that we misclassified discontinuation status and reasons for discontinuation based on chart review. We did not confirm endocrine therapy administration dates with pharmacy records or pill diaries, nor did we confirm reasons for discontinuation by patient report. Additionally, our analysis evaluated the association between each individual treatment-emergent symptom with discontinuation, but we did not evaluate whether the overall symptom burden affected the risk of discontinuation. Another limitation of our study is that we assessed pain with the PROMIS pain interference measure, a tool not specific to joint pain that may not have been sensitive enough to detect AI-associated musculoskeletal symptoms. It is possible that had we used a more sensitive and specific measure, we would have identified an association between treatment-emergent musculoskeletal pain and endocrine therapy discontinuation as has been reported previously. Additionally, we did not collect any data regarding comorbidities and, for concomitant medications, we only collected the number of medications (including both over the counter and prescribed medications) at baseline in our study, thus we cannot address any potential associations between specific medication classes and their side effects, nor the associations of any changes in concomitant medications during study participation or of any comorbidities, with time-to-discontinuation of endocrine therapy. Furthermore, our study population was predominantly white and high socioeconomic status which may limit generalizability of our findings to other populations.

Finally, it must be noted that while a change in a score of at least the MID is considered clinically significant, it is not certain that a change of this degree is truly the *minimal* change that is significant^48^. If anchored to endocrine therapy discontinuation, it is possible that smaller changes in scores would be clinically significant and had we looked at smaller changes, we may have identified associations between discontinuation and worsening of other symptoms as have previously been reported such as musculoskeletal discomfort, depression, and anxiety^10,12,23,24,26,32,37,40–42^. Further study is needed to determine the optimal PRO measures for symptom assessment during endocrine therapy and the score thresholds to best identify treatment-emergent symptoms associated with discontinuation in diverse populations.

The use of PROs to assess symptoms improves clinical outcomes including hospitalization rates, emergency room utilization, chemotherapy delivery and quality of life, effects likely mediated by enhanced symptom detection and subsequent symptom management^54,76^. To date, whether use of PROs impacts oral cancer therapy persistence has not been prospectively determined. In this real-world cohort of patients taking adjuvant endocrine therapy for early HR + breast cancer, we found that treatment-emergent endocrine symptoms and sleep disturbance detected via PRO measures were associated with early endocrine therapy discontinuation. This finding is proof of the principle that collecting serial PROs during routine clinical care can identify patients at risk for endocrine therapy discontinuation due to side effects who may benefit from symptom management interventions. To this end, we are prospectively evaluating the feasibility of using recommended symptom management pathways for patients receiving endocrine therapy who have symptoms identified on PROs. Future studies will also be needed to clarify the optimal timing, the optimal PRO domains and MIDs to use when implementing PRO collection to identify patients receiving endocrine therapy at risk for treatment discontinuation. Better symptom detection and management has the potential not only to help patients feel better, but also to enhance endocrine therapy persistence, an effect that could ultimately reduce breast cancer recurrence and mortality.

## Methods

### Study population

We enrolled women with HR + stage 0-III breast cancer-initiating adjuvant endocrine therapy with tamoxifen or an AI to a clinic-based prospective cohort between March 2012 and December 2016 at Johns Hopkins (ClinicalTrials.gov Identifier: NCT01937052, registered September 3, 2013). The study cohort was a convenience sample with candidate participants identified by screening medical oncology provider clinic schedules and by provider referral. Pre-menopausal women could receive concurrent OFS. Participants could enroll when first initiating endocrine therapy or upon switching from one agent to another. All participants signed written informed consent. The study was approved by the Johns Hopkins IRB.

### Discontinuation

Endocrine therapy discontinuation was defined as stopping the endocrine therapy initiated at enrollment prior to completing 5 years of treatment for at least 6 weeks due to side effects/intolerance or other reasons besides recurrence, new primary breast cancer, completion of at least 5 years of endocrine therapy or switching to another type of endocrine therapy after less than 6 weeks off therapy. Participants were allowed to continue endocrine therapy beyond 5 years. Endocrine therapy discontinuation and reason for discontinuation were determined by chart review. Reasons for discontinuation were classified as: completed treatment, discontinued due to distant metastases, discontinued due to loco-regional recurrence, discontinued due to side effects/intolerance, discontinued tamoxifen to transition to AI and discontinued due to other reasons. No patient discontinued due to a new primary breast cancer.

### Patient-reported outcomes

PROs were collected at baseline and 3, 6, 12, 24, 36, 48, and 60 months using the online PatientViewpoint website^77–79^. Measures included the Patient-Reported Outcome Measurement Information System (PROMIS) Version 1.0 pain interference, fatigue, depression, anxiety, physical function, and sleep disturbance short forms; the Endocrine Subscale of the Functional Assessment of Cancer Therapy - Endocrine Symptom (FACT-ES) measure; and the Medical Outcomes Study Sexual Problems (MOS-SP) scale^30,80–87^. Treatment-emergent symptoms were defined as worsening of scores at any time compared to baseline in increments meeting or exceeding the MID for each measure. PROMIS measures are scored with a T-score metric for which 50 represents the mean in the United States (US) population and 10 is the standard deviation (SD). Higher scores on PROMIS measures indicate more of the outcome measured. PROMIS measures have been validated in early-stage cancer patients with a MID of 3–5 points^80–83,88^. We considered the midpoint of this range (4 points) to be the MID. Endocrine Subscale FACT-ES scores range from 0–76 with lower scores indicating more endocrine symptoms. The mean and SD on the Endocrine Subscale of the FACT-ES in women with early breast cancer are 59 and 9.7 respectively^30^. In accordance with the distribution-based, Effect Size method of identifying a MID for a PRO measure, we used 0.5 SD to define a medium effect size as a conservative estimate of the MID on the Endocrine Subscale of the FACT-ES and rounded this estimate to 5 points^48^. MOS-SP scores range from 0–100 with higher scores indicating more sexual problems. The reported mean MOS-SP score for women with early breast cancer ranges from approximately 20–36 with SD approximately 27–31^28,85,87^. As has previously been done, due to some uncertainty about the mean and SD on the MOS-SP in the literature, we used the distribution-based, Empirical Rule Effect Size method to guide the identification of the MID for the MOS-SP. Based on this method, the SD was assumed to be one-sixth of 100 and the estimated MID was calculated by dividing this number by half, yielding an estimated MID of 8 points^28,89^. A summary of PRO scores was available to clinicians at the time of follow-up clinic visits.

### Statistical analysis

Clinico-demographic characteristics of the participants and PRO scores over time are presented descriptively using mean (SD), median (range or interquartile range), and proportions. We used Fisher’s exact test to compare categorical measures, *t*-test to compare means and Wilcoxon rank-sum test to compare medians between subgroups of the study population as appropriate. Mean changes in PRO scores in the first 24 months compared to baseline were estimated using a linear mixed-effects modeling approach with the PRO as the outcome, fixed effects for each time point, and a random intercept for each participant. A corresponding four-degree-of-freedom test was used to summarize the overall change in the first 24 months.

The time to discontinuation of endocrine therapy was calculated as the time from study enrollment to endocrine therapy discontinuation. Participants who stopped the endocrine therapy initiated at enrollment due to recurrence, new primary breast cancer, completion of at least 5 years of endocrine therapy or who switched to another type of endocrine therapy after less than 6 weeks interruption were censored. All other participants were censored at the date of the last clinic visit before the database was locked May 15, 2020. Time to discontinuation was estimated for the entire cohort and according to type of endocrine therapy (AI versus Tamoxifen) using the Kaplan-Meier method.

To assess how variables measured at baseline and during follow-up were associated with time to discontinuation, we fit Cox proportional hazards models using a time-dependent covariate structure. Non-time dependent demographic variables considered in the models included age at enrollment, neighborhood poverty rate, and race. Neighborhood poverty rate, the percentage of persons living in a zip code with a family income below the federal poverty line based on US census data, was used as a surrogate for socioeconomic status (SES) with >15% considered indicative of low SES^90^. Clinical characteristics in the models included stage, HER2 status, type of surgery, receipt of chemotherapy, receipt of radiation, number of self-reported concomitant medications at enrollment and type of endocrine therapy. Time-dependent covariates included change in PRO scores compared to baseline based on MIDs, specified as worsening of scores at any time up to 60 months on the PROMIS measures in 4-point increments, on the Endocrine Subscale of the FACT-ES in 5-point increments, and on the MOS-SP in 8-point increments. First, associations between variables with time to endocrine therapy discontinuation were estimated with univariate models. We then used a forward and backward stepwise selection approach based on Akaike’s Information Criterion (AIC) to identify a final multivariate Cox proportional hazards model for the association of treatment-emergent symptoms, assessed by minimal important changes in PRO scores up to 5 years, and clinico-demographic variables with time to endocrine therapy discontinuation^91,92^. Analyses were completed with R version 4.0.0^93^.

### Reporting summary

Further information on research design is available in the Nature Research Reporting Summary linked to this article.

## Supplementary information

Reporting Summary Checklist

## Data Availability

The data that support the findings of this study are not publicly available as they contain information that could compromise individual patient privacy, however they are available upon reasonable request from the corresponding author.
