# Supplementary material for: Association of treatment-emergent symptoms identified by patient-reported outcomes with adjuvant endocrine therapy discontinuation
Source: NPJ Breast Cancer. 2022 Apr 21;8:53. doi: 10.1038/s41523-022-00414-0 (PMC9023490; doi:10.1038/s41523-022-00414-0)
Supplement: Supplementary file 1 — Reporting Summary Checklist [file 41523_2022_414_MOESM1_ESM.pdf]

nature portfolio

Corresponding author(s): Karen Lisa Smith

Last updated by author(s): Feb 11, 2022

Reporting Summary

Nature Portfolio wishes to improve the reproducibility of the work that we publish. This form provides structure for consistency and transparency in reporting. For further information on Nature Portfolio policies, see our Editorial Policies and the Editorial Policy Checklist.

Statistics

For all statistical analyses, confirm that the following items are present in the figure legend, table legend, main text, or Methods section.

n/a	Confirmed	
☐	☒	The exact sample size (*n*) for each experimental group/condition, given as a discrete number and unit of measurement
☐	☒	A statement on whether measurements were taken from distinct samples or whether the same sample was measured repeatedly
☐	☒	The statistical test(s) used AND whether they are one- or two-sided<br>*Only common tests should be described solely by name; describe more complex techniques in the Methods section.*
☐	☒	A description of all covariates tested
☐	☒	A description of any assumptions or corrections, such as tests of normality and adjustment for multiple comparisons
☐	☒	A full description of the statistical parameters including central tendency (e.g. means) or other basic estimates (e.g. regression coefficient) AND variation (e.g. standard deviation) or associated estimates of uncertainty (e.g. confidence intervals)
☐	☒	For null hypothesis testing, the test statistic (e.g. *F*, *t*, *r*) with confidence intervals, effect sizes, degrees of freedom and *P* value noted<br>*Give P values as exact values whenever suitable.*
☒	☐	For Bayesian analysis, information on the choice of priors and Markov chain Monte Carlo settings
☒	☐	For hierarchical and complex designs, identification of the appropriate level for tests and full reporting of outcomes
☒	☐	Estimates of effect sizes (e.g. Cohen's *d*, Pearson's *r*), indicating how they were calculated

Our web collection on statistics for biologists contains articles on many of the points above.

Software and code

Policy information about availability of computer code

Data collection	PROs were collected on the PatientViewpoint interface
Data analysis	Analyses were completed with R version 4.0.0

For manuscripts utilizing custom algorithms or software that are central to the research but not yet described in published literature, software must be made available to editors and reviewers. We strongly encourage code deposition in a community repository (e.g. GitHub). See the Nature Portfolio guidelines for submitting code & software for further information.

Data

Policy information about availability of data

All manuscripts must include a data availability statement. This statement should provide the following information, where applicable:

- Accession codes, unique identifiers, or web links for publicly available datasets
- A description of any restrictions on data availability
- For clinical datasets or third party data, please ensure that the statement adheres to our policy

The data that support the findings of this study are not publicly available as they contain information that could compromise individual patient privacy, however they are available upon reasonable request from the corresponding author.

Field-specific reporting

Please select the one below that is the best fit for your research. If you are not sure, read the appropriate sections before making your selection.

☐ Life sciences ☒ Behavioural & social sciences ☐ Ecological, evolutionary & environmental sciences

For a reference copy of the document with all sections, see nature.com/documents/nr-reporting-summary-flat.pdf

Behavioural & social sciences study design

All studies must disclose on these points even when the disclosure is negative.

Study description	This was an observational cohort. Participants received routine care and completed questionnaires.
Research sample	Participants had breast cancer and were initiating adjuvant endocrine therapy.
Sampling strategy	There was no pre-defined sample size. This was a convenience sample of patients initiating adjuvant endocrine therapy. All patients initiating adjuvant endocirne therapy receiving breast cancer care at Johns Hopkins sites were eligible. Potential participants were identified by provider referral and/or by screening provider clinic schedules.
Data collection	Participants completed questionnaires using the online PatientViewpoint interface.
Timing	March 2012 through December 2016
Data exclusions	No exclusions
Non-participation	No participants dropped out. As discussed in the manuscript, some participants did not complete all the questionnaires.
Randomization	This was not a randomized study.

Reporting for specific materials, systems and methods

We require information from authors about some types of materials, experimental systems and methods used in many studies. Here, indicate whether each material, system or method listed is relevant to your study. If you are not sure if a list item applies to your research, read the appropriate section before selecting a response.

Materials & experimental systems

n/a	Involved in the study	
☒	☐	Antibodies
☒	☐	Eukaryotic cell lines
☒	☐	Palaeontology and archaeology
☒	☐	Animals and other organisms
☐	☒	Human research participants
☐	☒	Clinical data
☒	☐	Dual use research of concern

Methods

n/a	Involved in the study	
☒	☐	ChIP-seq
☒	☐	Flow cytometry
☒	☐	MRI-based neuroimaging

Human research participants

Policy information about studies involving human research participants

Population characteristics	see above
Recruitment	see above. Since this was a convenience sample, it is possible patients who were recruited differed from those who were not recruited.
Ethics oversight	The protocol was approved by the Johns Hopkins IRB.

Note that full information on the approval of the study protocol must also be provided in the manuscript.

Clinical data

Policy information about clinical studies

All manuscripts should comply with the ICMJE guidelines for publication of clinical research and a completed CONSORT checklist must be included with all submissions.

Clinical trial registration	ClinicalTrials.gov Identifier: NCT01937052
Study protocol	The full protocol is available upon reasonable request to the corresponding author
Data collection	Patients were recruited from Johns Hopkins breast medical oncology clinics from March 2012 through December 2016
Outcomes	outcomes included endocrine therapy discontinuation as determined by chart review and patient responses to the questionnaires.
